# Areca Nut Components Affect COX-2, Cyclin B1/cdc25C and Keratin Expression, PGE2 Production in Keratinocyte Is Related to Reactive Oxygen Species, CYP1A1, Src, EGFR and Ras Signaling

**DOI:** 10.1371/journal.pone.0101959

**Published:** 2014-07-22

**Authors:** Mei-Chi Chang, Yi-Jane Chen, Hsiao-Hua Chang, Chiu-Po Chan, Chien-Yang Yeh, Yin-Lin Wang, Ru-Hsiu Cheng, Liang-Jiunn Hahn, Jiiang-Huei Jeng

**Affiliations:** 1 Team of Biomedical Science, Chang-Gung University of Science and Technology, Kwei-Shan, Taoyuan, Taiwan; 2 Laboratory of Pharmacology, Toxicology and Chemical Carcinogenesis, School of Dentistry and Department of Dentistry, National Taiwan University Hospital; and National Taiwan University Medical College, Taipei, Taiwan; 3 Department of Dentistry, Chang Gung Memorial Hospital, Taipei, Taiwan; Taipei Medical University, Taiwan

## Abstract

**Aims:**

Chewing of betel quid (BQ) increases the risk of oral cancer and oral submucous fibrosis (OSF), possibly by BQ-induced toxicity and induction of inflammatory response in oral mucosa.

**Methods:**

Primary gingival keratinocytes (GK cells) were exposed to areca nut (AN) components with/without inhibitors. Cytotoxicity was measured by 3-(4,5-dimethyl- thiazol- 2-yl)-2,5-diphenyl-tetrazolium bromide (MTT) assay. mRNA and protein expression was evaluated by reverse transcriptase-polymerase chain reaction (RT-PCR) and western blotting. PGE_2_/PGF_2α_ production was measured by enzyme-linked immunosorbent assays.

**Results:**

Areca nut extract (ANE) stimulated PGE_2_/PGF_2α_ production, and upregulated the expression of cyclooxygenase-2 (COX-2), cytochrome P450 1A1 (CYP1A1) and hemeoxygenase-1 (HO-1), but inhibited expression of keratin 5/14, cyclinB1 and cdc25C in GK cells. ANE also activated epidermal growth factor receptor (EGFR), Src and Ras signaling pathways. ANE-induced COX-2, keratin 5, keratin 14 and cdc25C expression as well as PGE_2_ production were differentially regulated by α–naphthoflavone (a CYP 1A1/1A2 inhibitor), PD153035 (EGFR inhibitor), pp2 (Src inhibitor), and manumycin A (a Ras inhibitor). ANE-induced PGE_2_ production was suppressed by *piper betle* leaf (PBL) extract and hydroxychavicol (two major BQ components), dicoumarol (a NAD(P)H:Quinone Oxidoreductase - NQO1 inhibitor) and curcumin. ANE-induced cytotoxicity was inhibited by catalase and enhanced by dicoumarol, suggesting that AN components may contribute to the pathogenesis of OSF and oral cancer via induction of aberrant differentiation, cytotoxicity, COX-2 expression, and PGE_2_/PGF_2α_production.

**Conclusions:**

CYP4501A1, reactive oxygen species (ROS), EGFR, Src and Ras signaling pathways could all play a role in ANE-induced pathogenesis of oral cancer. Addition of PBL into BQ and curcumin consumption could inhibit the ANE-induced inflammatory response.

## Introduction

Oral leukoplakia, oral submucous fibrosis (OSF) and oral cancer are popular diseases in India, Taiwan, Sri Lanka and many other south-east Asian countries where betel quid (BQ) chewing is popular [1]–[3]. Oral cancer has been the 4th cancer death reason in the male of Taiwan. BQ is considered to be one major contributing factor. BQ contains mainly areca nut (AN), inflorescence *piper betle*, lime with/without *piper betle* leaf (PBL) or tobacco [2]. However, the precise mechanisms are still not clear. Chemical carcinogenesis is a multi-step processes including initiation, promotion and progression, where genetic (DNA damage) and epigenetic alterations (histone acetylation, tissue inflammation etc.) are involved [2], [4]. Some chemical carcinogens should be metabolically activated to direct-acting electrophiles or generation of reactive oxygen species (ROS) by cytochrome P450 (CYP) or other phase 1 enzymes prior to reacting with DNA [4]. ROS production and tissue inflammation may further contribute to the carcinogenic processes by inducing more DNA damage, cell cycle arrest, aberrant differentiation, changes of signal transduction pathways, and thereby OSF and clinical tumors as observed in BQ chewers [5]. Moreover, epidermal growth factor receptor (EGFR), Src and Ras activation are possible molecular factors for chemical carcinogenesis [6]–[8]. However, their roles in the pathogenesis of BQ chewing-related oral mucosal diseases are still obscure.

EGFR (HER1, erbB1) is a receptor tyrosine kinase (RTK) that regulates the cell proliferation and differentiation via Src, Ras or phosphoinositide 3-kinase (PI3K)/protein kinase B (AKT) signaling. Recently, EGFR expression, activation and downstream k-Ras as well as mitogen-activated protein kinase (MAPK) signaling are shown to be involved in the pathogenesis oral cancer [6], [8]. Src is a non-receptor tyrosine kinase that can be activated by metals, ROS and UV irradiation [7]. Activated Src and Ras may induce downstream signaling of MAPK, nuclear factor kappa B (NF-κB) and PI3K [8]. Accumulating evidence indicates that ROS generated during metabolism of toxic chemicals may activate receptors, receptor-activated protein kinases and nuclear transcription factors, including growth factor receptors, Src kinase, Ras signaling, MAPKs, PI3K/Akt pathway, NF-κB, activator protein 1, p53 etc [7], [8]. Signaling of these pathways by ROS may mediate global cellular effects including DNA/cell damage, inflammation, cell cycle regulation, apoptosis and gene expression [7]. Excessive ROS production may also cause lipid peroxidation, protein modification and DNA damage. Interestingly, exposure to BQ has been shown to induce ROS production *in vitro* and *in vivo* and MAPK activation [2], [9], implicating its role in the activation of upstream EGFR, Src and Ras signaling in oral mucosal cells.

Cycloxygenase-2 (COX-2) expression and prostanoids production may regulate inflammatory responses such as vasodilatation, increase of vascular permeability, stimulation of inflammatory cell infiltration that are popularly noted in oral mucosa of oral cancer and OSF [2]. An elevated expression of COX-2 in oral cancer with different stages has been reported [10]. Tissue inflammation has been shown to play critical role in multistage chemical carcinogenesis via generation of DNA-damaging ROS by inflammatory cells, suppression of immune defense, stimulation of lipid peroxidation, angiogenesis, cell proliferation, tumor invasion and metastasis [11]. Previous reports have found the induction of COX-2 and PGE_2_ production of gingival keratinocytes (GK) by AN extract (ANE) via activation of MEK/ERK [12]. Whether EGFR, Src and Ras are important in this event awaits further investigation, because the mutation and elevated expression of CYP, COX2, EGFR, Src and Ras in oral cancer and precancer are reported [6], [8], [13], [14]. Moreover, PBL and its phenolic – hydroxychavicol (HC) exhibit antioxidative property, but is also reported to posses potential oxidative stress [15], [16]. Clinically one critical health issue is whether addition of PBL into BQ and consumption of ginger/curry may enhance or attenuate the carcinogenicity of BQ.

To further understand the chemical carcinogenesis of BQ chewing-related cancer, we proposed that BQ can be metabolized by CYP to generate ROS and activate EGFR, Src, and Ras signaling pathways to stimulate COX-2 expression, prostanoids production and affect the differentiation of GK. The results of this study may highlight our knowledge on the pathogenesis of BQ-related cancer and is useful for future chemoprevention and targeting therapy of BQ chewing-related diseases.

## Materials and Methods

### Materials

Keratinocyte growth medium (KGM-SFM), pituitary gland extract and epidermal growth factor (EGF) etc. were from Gibco (Life Technologies, BRL, Grand Island, NY, USA). Catechol, arecoline, PD153035, manumycin A, pp2, curcumin, catalase, α-naphthoflavone, Zn-protoporphyrin dicoumarol and 3-(4,5-dimethyl-thiazol- 2-yl)-2,5-diphenyl-tetrazolium bromide (MTT) were obtained from Sigma (Sigma Chemical Company, St. Louis, MO, USA). PGE_2_ and PGF_2α_ ELISA kits were purchased from Cayman Chemical Company (Ann Arbor, MI, USA). Reagents for reverse transcription (RT) and polymerase chain reaction (PCR) were purchased from Invitrogen (Invitrogen Corporation, Carlsbad, CA, USA). Total RNA isolation kits were from Macherey-Nagel (Macherey-Nagel Inc., Easton, PA, USA). ANE and PBL extract were prepared and weighed as previously [12], [15]. Hydroxychavicol (HC) was synthesized and characterized as before [16]. Specific PCR primer sets for COX-2, CYP450 isoforms, hemeoxygenase-1 (HO-1), cyclinB1, cdc25C, cdc2, keratin 5, keratin 14 and β-actin were synthesized by Genemed Biotechnologies, Inc. (San Francisco, CA, USA). Pathscan p-Src (Y416) and p-EGFR (Tyr845) ELISA kits were from Cell Signaling (Cell Signaling Technology Inc., MA, USA). EZ-Detect™ Ras activation assay kits were from Pierce (Rockford, IL, USA). Mouse anti-human COX-2 and anti-GAPDH antibody were from Santa Cruz.

### Culture of Gingival Keratinocytes (GK)

GK were cultured as described previously [12]. Clinically human gingiva (with a gingivitis index <1) was acquired during crown-lengthening procedures with written informed consent by the patients and approval by the Ethics Committee, National Taiwan University Hospital. The individual in this manuscript has given written informed consent to publish these case details. Most of the subepithelial connective tissue was removed first by a surgical knife and then gingival tissues were cut into small pieces, placed onto culture dishes in KGM-SFM with supplements. The cell passages ranging from 1 to 3 were used for this study.

### Effect of ANE on Prostanoids Production of GK

Near confluent GK in 6-well culture plates were exposed to 2 ml fresh medium containing various concentrations of ANE, catechol or PBL extract. Cells were further incubated for 24 h. Culture medium was collected for analysis of PGE_2_ and PGF_2α_ by ELISA.

### Effect of ANE on EGFR and Src Activation in GK

Near confluent GK were exposed to ANE (800 µg/ml) for different time periods (0–120 min). Cells were washed with phosphate-buffered saline (PBS) and then cell lysates were prepared by dissolving cells in lysis buffer (20 mM Tris (pH 7.5), 150 mM NaCl, 1 mM ethylene diaminetetraacetate (EDTA), 1 mM ethylene glycolbis (2-aminoethyl)-N,N,N',N'-tetraacetic acid (EGTA), 1% Triton X-100, 2.5 mM sodium pyrophosphate, 1 mM β-glycerolphosphate, 1 mM Na3VO4, 1 µg/ml leupeptin. Protein concentrations were determined by BioRad protein assay kit. Equal amounts of protein (600 µg) were used for analysis of Src and EGFR activation by Pathscan p-Src (Y416) and p-EGFR (Tyr845) ELISA according to the manufacture's instruction [17].

### Effects of ANE on Ras Activation of GK

Activation of Ras in GK by AN extract was measured using the EZ-Detect™ Ras activation assay kits as described previously [18]. Near confluent GK were exposed to AN extract (800 µg/ml) for 0, 30, 60 and 120 sec. Cell lysates were collected and 500 µg of proteins were incubated with 80 µg GST-Raf1-RBD and SwellGel Immobilized Glutathione Disc in a rotor at 4°C for 1 h. Cell lysates that were preincubated with 10 mM GTPγS and used as positive control. The active Ras-GTP was pulled down from the cell lysates by centrifugation at 10000 rpm for 1 min and then subjected to electrophoresis and transferred to polyvinylidene difluoride (PVDF) membrane. The membrane was soaked in blocking reagent (20 mM Tris, pH 7.4; 125 mM NaCl; 0.2% Tween 20; 5% nonfat dry milk; and 0.1% sodium azide) at room temperature for 30 min and incubated for 2 h with anti-Ras antibody. Membranes were exposed to secondary antibody, washed and the immuno-reactive bands were detected on Fuji X-ray film by Enhanced Chemiluminescence (ECL) reagents.

### Effect of Various Inhibitors on the ANE-induced PGE_2_ Production of GK and the Concomitant Cytotoxicity

Near confluent GK were exposed to fresh medium containing ANE (800 µg/ml) with/without inhibitors (catalase, α-naphthoflavone, Zn-protoporphyrin, PD153035, pp2, manumycin, PBL extract, HC, curcumin or dicoumarol) for 24 hr. Inhibitors were added 30 min before ANE and then co-incubated for 24 hr. Culture medium was collected for measurement of PGE_2_ production by enzyme-linked immunosorbant assay (ELISA). Results were expressed as concentration of PGE_2_ and PGF_2α_ (pg/ml). In some experiments, cell layers were washed by PBS and cytotoxicity was measured by MTT assay as before [12]. Briefly, cell layers were incubated with fresh medium containing 0.5 mg/ml of MTT for 2 h. The produced formazan was dissolved in 2 ml of dimethylsulfoxide (DMSO) and read at OD540 with a Dynatech Microwell plate reader.

### Effect of ANE on COX-2, CYP1A1, Keratins and Cell Cycle-related Genes Expression in GK and its Modulation by Catalase, α-naphthoflavone, Zn-protoporphyrin, PD153035, pp2, Manumycin A, Aspirin, PBL Extract or HC

Near confluent GK were incubated in KGM-SFM containing ANE (100–800 µg/ml) for 24-hrs. Cell layers were washed with PBS, dissolved and used for RNA isolation by RNA isolation kits. In some experiments, GK were exposed to ANE with/without inhibitors (as indicated) for 24 h. Inhibitors were added 30 min before addition of ANE and then co-incubated.

*Semi-quantitative Reverse-transcriptase and Polymerase Chain Reaction (RT-PCR)* [12] - Briefly, 3 µg of denatured total RNA was reverse transcribed in a total volume of 80 µl reaction mixture containing 8 µl of random hexamer, 8 µl of dNTP (10 mM), RNA and DEPC water at 65°C for 5 min and more than 2 min at 4°C. Then 80 µl of cDNA synthesis mixure (by mixing 104 µl of 10× RT buffer, 104 µl 0.1 M Dithiothreitol [DTT], 208 µl 25 mM MgCl_2_, 28 µl of RNase OUT™, 28 µl SuperScript™ III RT and 8 µl DEPC water) were added into above tubes. The reaction mixtures were allowed for incubation at 25°C for 10 min, 50°C for 50 min and 85°C for 5 min. Finally, 1 µl of RNase H was added and incubated at 37°C for 20 min.

Five microliters of cDNA were used for PCR amplification in 50 µl reaction mixture containing 5 µl of 10× TAQ buffer, 4 µl of dNTP (2.5 mM), 1 µl of each specific primer, and 0.2 µl of Super TAQ enzyme (2 U/ µl). They were heated to 94°C for 5 minutes in the first cycle, then amplified for 15–35 cycles of 94°C for 1 min, 55°C for 1 min and then 72°C for 2 min with a thermal cycler (Perkin Elmer 4800, PE Applied Biosystems, Foster city, CA, USA). Finally, the reaction was terminated at 72°C for 10 minutes. The sequence of primer pairs was shown in **Table 1** [19]–[22]. The amplified DNA products were loaded into a 1.8% of agarose gel in 1× of Tris/Borate/EDTA (TBE) buffer for electrophoresis. Gels were stained with ethidium bromide, photographed under UV camera and AlphaEaseFC software program.

**Table 1:** PCR primers used in this study and the size of generated products.

Name of primers	Primer sequence	Primer sequence	expected product	Reference
β-actin	AAGAGAGGCATCCTCACCCT	TACATGGCTGGGGTGTTGAA	218 bp	Chang et al. (2010) [20]
COX-2	TTCAAATGAGATTGTGGGAAAATTGCT	AGATCATCTCTGCCTGAGTATCTT	305 bp	Chang et al. (2010)
CYP1A1	TCACAGACAGCCTGATTGAG	GATGGGTTGACCCATAGCTT	432 bp	Hakkola et al. (1996) [19]
CYP1A2	TGGCTTCTACATCCCCAAGAAAT	TTCATGGTCAGCCCGTAGAT	308 bp	Hakkola et al. (1996)
CYP2B6/B7	CCATACACAGAGGCAGTCAT	GGTGTCAGATCGATGTCTTC	356 bp	Hakkola et al. (1996)
CYP2A6/A7	GTGTGGACATGATGCCGT	AGGACTTGAGGCGGAAGT	1151 bp	Hakkola et al. (1996)
CYP2C8/19	GCTAAAGTCCAGGAAGAGATTGA	TCCTGCTGAGAAAGGCATGAAGT	332 bp	Hakkola et al. (1996)
CYP2E1	AGCACAACTCTGAGATATGG	ATAGTCACTGTACTTGAACT	365 bp	Hakkola et al. (1996)
CYP2F1	ATGAACTTGCCGCACCGCGT	AGCGAAAAGCTCTGCAGGAT	283 bp	Hakkola et al. (1996)
CYP3A3/A4	CCAAGCTATGCTCTTCACCG	TCAGGCTCCACTTACGGTGC	323 bp	Hakkola et al. (1996)
CYP3A5	TGTCCAGCAGAAACTGCAAA	TTGAAGAAGTCCTTGCGTGTC	470 bp	Hakkola et al. (1996)
CYP3A7	CTATGATACTGTGCTACAGT	TCAGGCTCCACTTACGGTCT	474 bp	Hakkola et al. (1996)
HO-1	AAGATTGCCCAGAAAGCCCTGGAC	AAGATTGCCCAGAAAGCCCTGGAC	399 bp	Chang et al. (2010) [20]
Cdc2	GGGGATTCAGAAATTGATCA	GGGGATTCAGAAATTGATCA	288 bp	Chang et al. (2010)
Cdc25C	CCTGGTGAGCCTTCGAAGACC	GCAGATGAAGTACACATTGCATC	456 bp	Chang et al. (2010)
Cyclin B1	AAGAGCTTTAAACTTTGGTCTGGG	CTTTGTAAGTCCTTGATTTACCATG	317 bp	Chang et al. (2010)
Keratin 5	TGGTCTCCCGTGCCGCAGTTCTAT	ATTTGGGATTGGGGTGGGGATTCT	139 bp	Endres et al. (2005) [21]
Keratin 14	TTCTCACAGCCACAGTGGAC	CATTGACATCTCCACCCACC	281 bp	Brown et al. (1994) [22]

### Effect of ANE on COX-2 Protein Expression and Src (Y416) Phosphorylation in GK – Western Blotting Analysis

Near confluent GK were exposed to ANE for different time points. Cell lysates was prepared as described before using lysis buffer (10 mM Tris-HCl, pH 7; 140 mM sodium chloride; 3 mM MgCl_2_; 0.5% NP-40; 2 mM phenylmethylsulfonyl fluoride; 1% aprotinin; and 5 mM DTT) [12]. The concentration of protein was determined by Bio-Rad protein assay kits. Proteins (50 µg/lane) were loaded for 12% SDS-polyacrylamide gel electrophoresis (Scie-Plas, UK) and then transferred to PVDF membrane for electroblotting. The membranes were blocked for 30 min at room temperature in a blocking reagent (20 mM Tris, pH 7.4; 125 mM NaCl; 0.2% Tween 20; 5% nonfat dry milk; and 0.1% sodium azide) and incubated for 2 h with mouse anti-human phospho-Src (Y416), anti-COX-2 and anti-glyceraldehyde-3-phosphate dehydrogenase (GAPDH) antibodies. Membranes were rinsed three times with TBST (10 mM Tris, pH 7.5; 100 mM NaCl, 0.1% Tween-20) for 10 min each, and then incubated with goat anti-mouse secondary antibody for 1 h. The membranes were washed 4 times with TBST and the immuno-reactive bands were developed by ECL reagent and visualized on Fuji X-ray film.

### Statistical Analysis

Four or more separate experiments were performed. Results were expressed as Mean ± SE and analyzed by one-way ANOVA and post hoc Tukey test. A p value <0.05 was considered to have statistically significant difference between 2 study groups.

## Results

**Figure 1:**
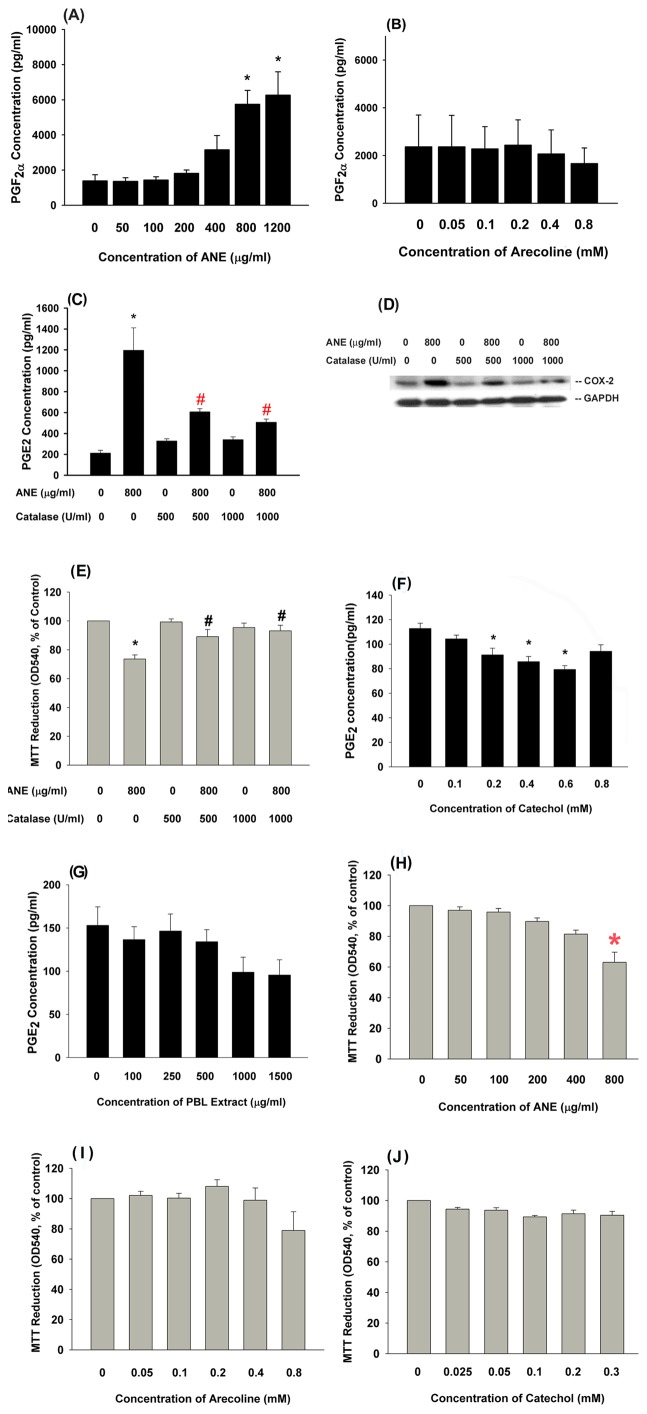
Effect of AN components on cytotoxicity, COX-2 expression and prostanoids production of GK and its modulation by catalase and PBL extract. (**A**) Stimulation of PGF_2α_ production of GK by ANE (50–1200 µg/ml) (n = 9). (**B**) Effect of arecoline on PGF_2α_ production of GK (n = 3), (**C**) The regulation of ANE-induced PGE_2_ production of GK by catalase (500 and 1000 U/ml) (n = 11), (**D**) Catalase inhibited the ANE-induced COX-2 protein expression in GK as analyzed by western blotting. One representative western blotting picture was shown. (**E**) Catalase attenuated the ANE-induced cytotoxicity to GK (n = 7). (**F**) Effect of catechol on PGE_2_ production of GK (n = 4), (**G**) Effect of PBL extract on PGE_2_ production of GK (n = 3). Results were expressed as concentration (pg/ml) in the culture medium. (**H**) Cytotoxicity of ANE to GK (n = 6), (**I**) Cytotoxicity of arecoline to GK (n = 4), (**J**) Cytotoxicity of catechol to GK (n = 3). Results were expressed as MTT reduction (% of control, Mean ± SE). *denotes significant difference when compared with control. #denotes statistical significant difference when compared with ANE (800 µg/ml) solely-treated group (P<0.05).

**Effect of BQ Components on the Cytotoxicity and Prostanoids Production of GK** Retraction and vacuoles formation of GK were noted after exposure to ANE for 24-h. ANE stimulates COX-2 expression and PGE_2_ production [12]. We further found that ANE induced PGF_2α_ production in GK (**Figure 1A**), but arecoline lacked of this effect (**Figure 1B**). Since ROS are shown to mediate the ANE genotoxicity and cytotoxicity to different cells [2], we tested and found that catalase (500 and 1000 U/ml) pretreatment and co-incubation attenuated the ANE-induced PGE_2_ production in GK (**Figure 1C**). Accordingly, catalase inhibited the ANE-induced COX-2 protein expression in GK (**Figure 1D**). Catalase was able to prevent the ANE-induced cytotoxicity (**Figure 1E**). The other BQ components, catechol (0.1–0.8 mM) and PBL extract (100–1500 µg/ml), showed little stimulatory effect on PGE_2_ production in GK and even inhibited basal level of PGE_2_ production (**Figure 1F, 1G**).

### Cytotoxicity of ANE, Arecoline and Catechol on GK

ANE showed mild cytotoxicity to GK (63% of control) at a concentration of 800 µg/ml (**Figure 1H**). Arecoline slightly decreased the viable cell number at a concentration of 0.8 mM (P>0.05) (**Figure 1I**). However, catechol showed little cytotoxicity to GK at concentrations of 0.1–0.3 mM (**Figure 1J**).

### Role of CYP1A1/1A2 and HO-1 in ANE-induced PGE2 Production and COX-2 Expression in GK

**Figure 2:**
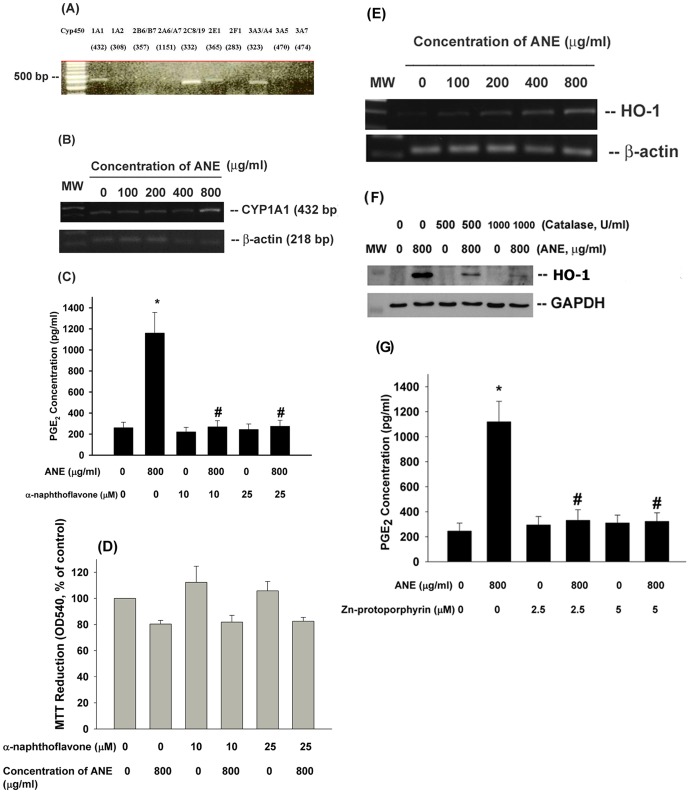
Role of cytochrome p450 and HO-1 on ANE-induced cytotoxicity and PGE2 production in GK. (**A**) Expression of cytochrome p450 isoforms (1A1, 1A2, 2B6/B7, 2A6/A7, 2C8/19, 2E1, 2F1, 3A3, 3A4, 3A5, 3A7) in cultured GK. One representative RT-PCR picture was shown, (**B**) Stimulation of CYP1A1 mRNA expression in GK by ANE was noted as revealed by RT-PCR (**C**) α–naphthoflavone (10 and 25 µM) pretreatment and co-incubation prevented the ANE-induced PGE_2_ production in GK (n = 10). (**D**) Effect of α–naphthoflavone on ANE-induced cytotoxicity to GK (n = 7), (**E**) Effect of ANE on HO-1 gene expression of GK, (**F**) Effect of catalase on ANE-induced HO-1 gene expression of GK, (**G**) Effect of Zn-protoporphyrin on ANE-induced PGE2 production in GK. *denotes significant difference when compared with solvent control. #denotes statistical significant difference when compared with ANE (800 µg/ml) solely-treated group (P<0.05).

ROS is generated by auto-oxidation of ANE or during metabolic activation of pro-carcinogens by CYPs [9]. We detected that GK expressed CYP1A1, 2C8/19, 2E1, 3A3/3A4 as analyzed by RT-PCR. But no PCR product was generated by using primers of CYP1A2, 2B6/B7, 2A6/A7, 2F1, 3A5 and 3A7 (**Figure 2A**). Exposure of GK to ANE (800 µg/ml) stimulated CYP1A1 mRNA expression after 24-hr of exposure (**Figure 2B**). Interestingly, α-naphthoflavone (10 and 25 µM), a CYP1A1/1A2 inhibitor, attenuated the ANE-induced PGE_2_ production in GK (**Figure 2C**). However, α–naphthflavone showed little preventive effect on ANE cytotoxicity as analyzed by MTT assay (**Figure 2D**). HO-1 as an oxidative stress response gene has been shown to have tissue protective effect, to resolve inflammation and may control tissue homeostasis [23]. We found that ANE stimulated HO-1 expression in GK (**Figure 2E**) and this event was attenuated by catalase (**Figure 2F**). Interestingly, inhibition of HO-1 by Zn-protoporphyrin (2.5 and 5 µM) decreased the ANE-induced PGE_2_ production (**Figure 2G**). This is partly due to cytotoxicity, because Zn-protoporphyrin partially enhanced the ANE-induced cytotoxicity (**data not shown**).

### Effect of EGFR Activation on ANE-induced COX-2 Expression and PGE_2_ Production in GK

**Figure 3:**
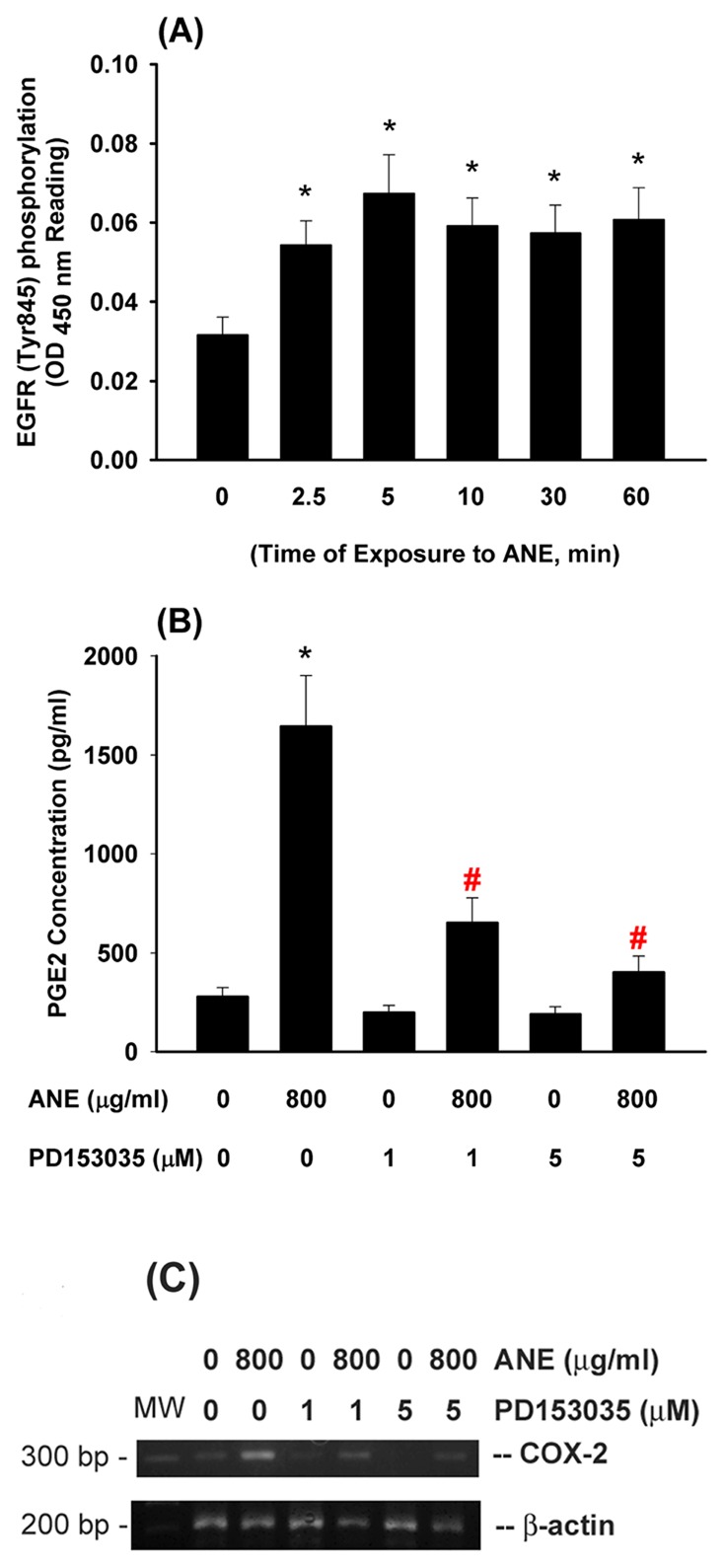
Effect of ANE on EGFR activation and its role in ANE-induced COX-2 expression and PGE2 production of GK. (**A**) AN extract (ANE) stimulated EGFR (Tyr845) phorphorylation within 60 min of exposure as analyzed by Pathscan p-EGFR ELISA. *denotes significant difference when compared with solvent control. (n = 10). (**B**) Pretreatment by PD153035 (1 and 5 µM, an EGFR antagonist) markedly attenuated the ANE-induced PGE_2_ production in GK. (**C**) Effect of PD153035 on ANE-induced COX-2 expression in GK. One representative PCR result was shown. *denotes significant difference when compared with solvent control. #denotes significant difference when compared with ANE (800 µg/ml)-treated group.

ANE (800 µg/ml) stimulated EGFR (Tyr845) phosphorylation as noted after 2.5 min of exposure and prolonged for more than 60 min (**Figure 3A**). Pretreatment and co-incubation by non-toxic concentration of PD153035 (1 and 5 µM, an EGFR inhibitor) inhibited the ANE-induced PGE_2_ production (**Figure 3B**). Accordingly, PD153035 also attenuated the ANE-induced COX-2 expression in GK (**Figure 3C**).

### Activation of Src by ANE and Its Role in Mediating COX-2 Expression and PGE_2_ Production in GK

**Figure 4:**
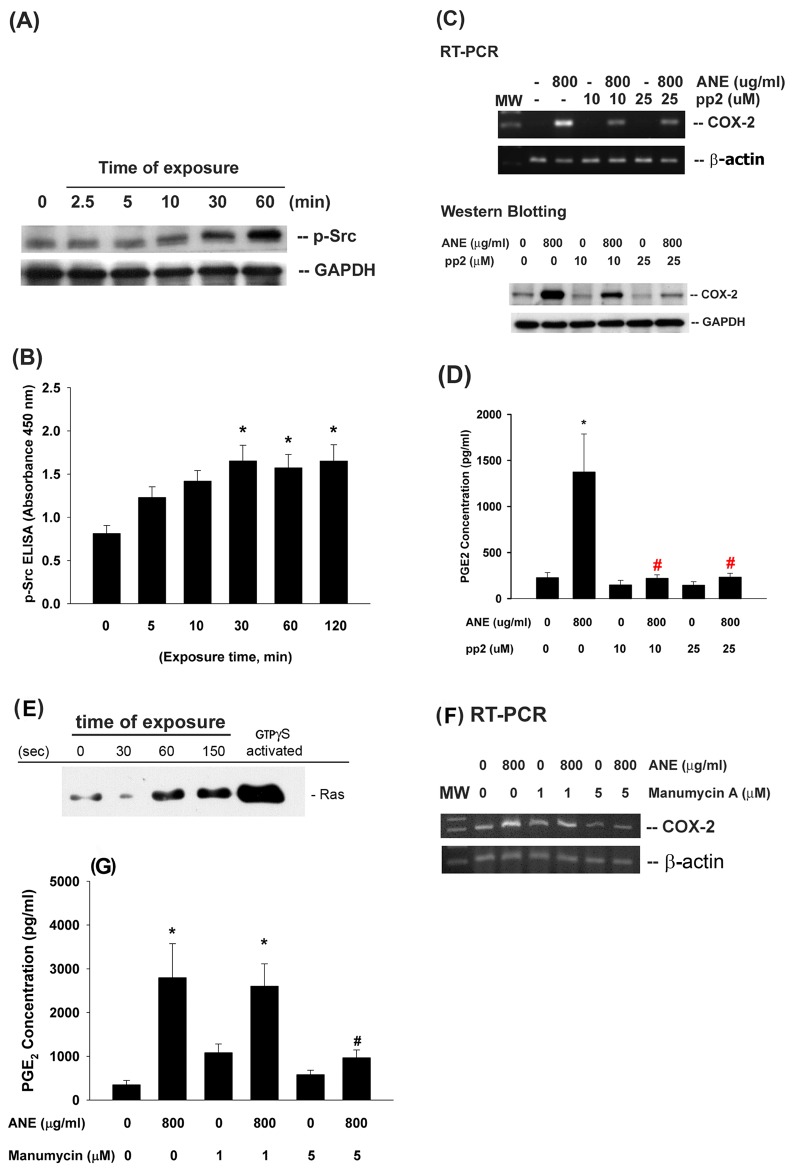
Effect of ANE on Src and Ras activation and its role in ANE-induced COX-2 expression and PGE2 production of GK. (**A**) GK were exposed to ANE (800 µg/ml) for different time points. Cell lysates were collected and used for analysis of Src phosphorylation (p-Src) by pathscan p-ELISA. Results were expressed as absorbance (OD450) (n = 9). *denotes significant difference when compared with control. (**B**) Inhibition of ANE-induced Src phosphorylation in GK by pp2, (**C**) pp2 inhibited the ANE-induced COX-2 mRNA and protein expression in GK. One representative PCR and western blotting result was shown. (**D**) pp2 prevented the ANE-induced PGE_2_ production in GK (n = 5). (**E**) ANE induced the activation of Ras in GK. GK were exposed to ANE (800 µg/ml) for different time points (30–150 secs). Cell lysates were collected and used for analysis of Ras activation using Ras activation assay kit (Pierce). GTPγS-activated was the positive control. (**F**) Manumycin A (1 and 5 µM) effectively prevented the ANE-induced COX-2 expression of GK, (**G**) Manumycin A also attenuated the ANE-induced PGE2 production of GK. *denotes significant difference when compared with solvent control. #denotes statistical significant difference when compared with ANE (800 µg/ml) solely-treated group (P<0.05)

ANE (800 µg/ml) stimulated Src activation, beginning after 10 min of exposure and showed significant difference during 30–120 min of exposure (**Figure 4A, 4B**). Intriguingly, inhibition of Src by pp2 (10 and 25 µM) effectively suppressed the ANE-induced COX-2 mRNA and protein expression (**Figure 4C**). Accordingly, pp2 also decreased ANE-induced PGE_2_ production in GK (**Figure 4D**). No marked enhancement or prevention of ANE cytotoxicity by pp2 was noted in these experimental conditions **(data not shown)**.

### Activation of Ras by ANE and Its Role in Mediating COX-2 Expression and PGE_2_ Production in GK

ANE (800 µg/ml) stimulated Ras activation of GK within short period of exposure (**Figure 4E**). Inhibition of Ras by manumycin A (1 and 5 µM) inhibited the ANE-induced COX-2 expression in GK as analyzed by RT-PCR (**Figure 4F**). Pretreatment and co-incubation by manumycin A (1 and 5 µM) also decreased the ANE-induced PGE_2_ production in GK (**Figure 4G**).

### Effect of Various Inhibitors on the ANE-induced Changes in Cdc2, Cdc25C, CyclinB1, Keratin 5 and Keratin 14 Expression of GK

**Figure 5:**
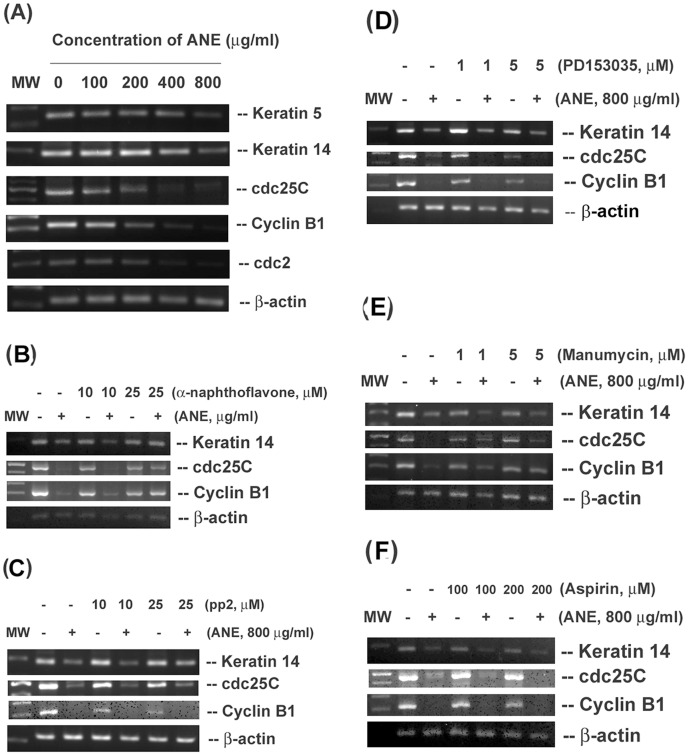
Effect of ANE on the cell cycle- and differentiation-related genes expression of GK and its modulation by various signal transduction inhibitors. (**A**) Effect of different concentrations of ANE on cell cycle- and differentiation-related genes expression. Effect of (**B**), α-naphthoflavone, (**C**) pp2, (**D**) PD153035, (**E**) manumycin A and (**F**) aspirin on the ANE-induced changes of keratin 14, cyclin B1 and cdc25C gene expression of GK. One representative RT-PCR picture was shown.

Exposure to ANE inhibited the cdc2, cdc25C, cyclin B1, keratin 5 and keratin 14 expression of GK (**Figure 5A**). We further evaluated the effect of various inhibitors on the ANE-induced change of keratin 14 (a differentiation marker), cdc25C and cyclin B1 expression. Interestingly α-naphthoflavone (10 and 25 µM) prevented the ANE-induced decrease of keratin 14, cyclin B1 and cdc25C expression (**Figure 5B**). The pp2 alone slightly inhibited cdc25C and cyclin B1 expression. However, pp2 pretreatment and co-incubation attenuated the ANE-induced decline of keratin 14 expression (**Figure 5C**). PD153035 showed little preventive effect toward the ANE-induced suppression of keratin 14, cdc25C and cyclin B1 (**Figure 5D**). On the other hand, manumycin A could not prevent the ANE-induced decline of keratin 14 expression. But manumycin A reversed the ANE-induced decrease in cyclin B1 and cdc25C expression (**Figure 5E**). Aspirin, a COX inhibitor, was not able to prevent the ANE-induced decline of cyclin B1, cdc25C and keratin 14 expression (**Figure 5F**).

### Effect of PBL Extract and HC on ANE-induced COX-2 Expression and PGE2 Production in GK

**Figure 6:**
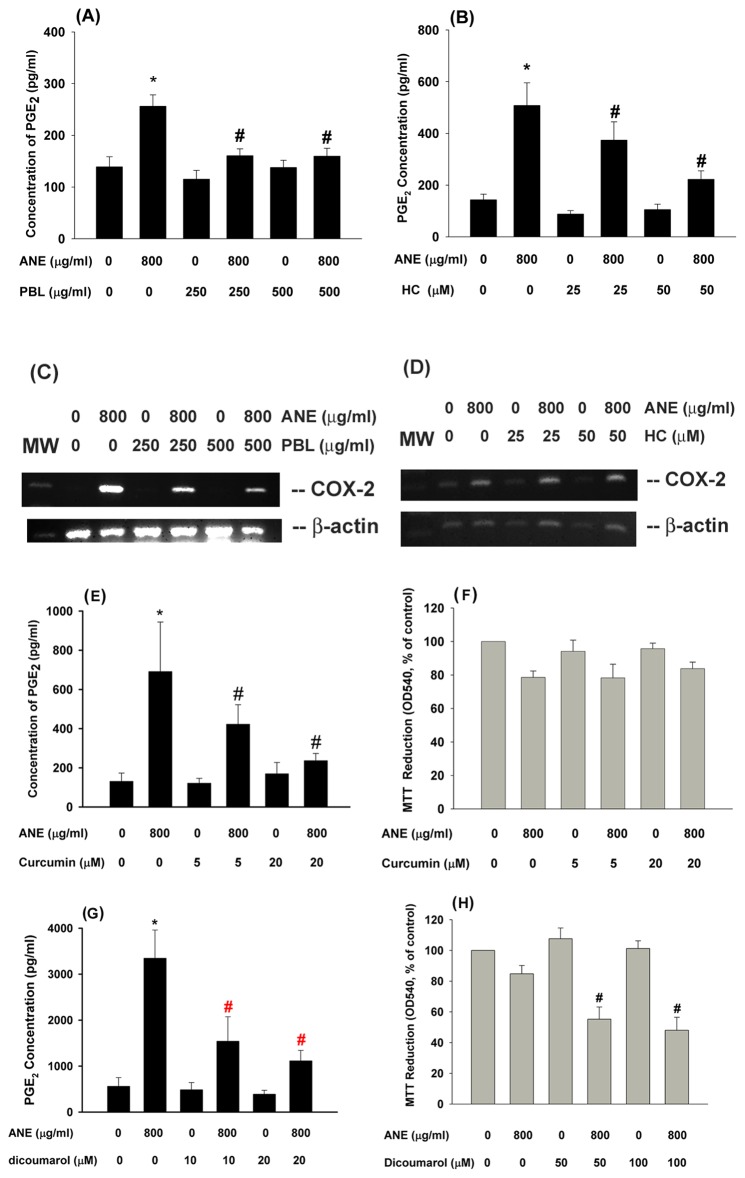
Effect of PBL and HC on the ANE-induced PGE_2_ production and Cox-2 expression of GK. (A) Effect of PBL (250 and 500 µg/ml) on ANE-induced PGE_2_ production in GK, (B) Effect of HC (25 and 50 µM) on ANE-induced PGE_2_ production in GK, (C) Effect of PBL on ANE-induced Cox-2 expression in GK, (D) Effect of HC (25 and 50 µM) on ANE-induced Cox-2 expression in GK. One representative PCR picture was shown. The expression of β-actin was used as control. (E) Effect of curcumin on ANE-induced PGE_2_ production in GK (n = 7). (F) Effect of curcumin on ANE-induced cytotoxicity as analyzed by MTT assay. Results were expressed as % of control (as 100%), Mean ± SE (n = 4). (G) Effect of dicoumarol (10 and 20 µM) on ANE-induced PGE_2_ production in GK (n = 6). (H) Effect of dicoumarol (50 and 100 µM) on ANE-induced cytotoxicity as analyzed by MTT assay (n = 5). *denotes statistically significant difference when compared with solvent control group, #denotes significant difference when compared with ANE (800 µg/ml)-treated group.

One critical health issue is whether addition of PBL into BQ may enhance or attenuate the BQ toxicity. We tested and found that PBL alone could slightly inhibit the PGE_2_ production of GK **(data not shown)**. In addition, PBL extract (250 and 500 µg/ml) inhibited the ANE-induced PGE_2_ production in GK (**Figure 6A**). At non-cytotoxic concentrations, HC (25 and 50 µM) as a PBL component also suppressed the ANE-induced PGE_2_ production in GK (**Figure 6B**). Similarly, PBL markedly inhibited the ANE-induced COX-2 mRNA expression (**Figure 6C**). But the inhibitory effect of HC (25 and 50 µM) on the COX-2 expression of GK was not so evident (**Figure 6D**).

### Effect of Curcumin and Dicoumarol on ANE-induced PGE2 Production in GK –

To know the preventive strategy for BQ-chewing-related diseases, we tested and found that curcumin (5 and 20 µM), a plant phenolic in tumeric and curry with antioxidant and anti-inflammatory properties [24] and popularly consumed in Taiwan and India, was shown to inhibit ANE-induced PGE_2_ production in GK (**Figure 6E**). However, curcumin cannot prevent the ANE-induced cytotoxicity (P>0.05) (**Figure 6F**).

GK was shown to express NADPH:quinone oxidoreductase (NQO-1, also as DT-diaphorase) mRNA and protein **(data not shown)**. Dicoumarol is a natural plant and fungal product with vitamin K antagonist (anticoagulant), NQO-1 inhibitor and NF-kB inhibitor properties. At non-cytotoxic concentrations (10 and 20 µM), dicoumarol was shown to inhibit the ANE-induced PGE_2_ production of GK (**Figure 6G**). Dicoumarol (50 and 100 µM) by itself showed little cytotoxicity to GK. However, pretreatment and co-incubation with dicoumarol (50 and 100 µM) increased the ANE-induced cytotoxicity to GK (**Figure 6H**).

## Discussion

BQ chewing is one major risk factor of oral cancer and OSF. Elevated COX-2 expression and prostanoids production is popularly observed in oral cancer and precancer tissues [10]. AN components are shown to induce tissue injury and inflammation, COX-2 expression and PGE_2_ production in GK [12]. We further found the stimulation of PGF_2α_ production of GK by ANE. This may explain why inflammatory cells infiltration is popularly noted in OSF and oral squamous cell carcinoma (OSCC) tissues [2]. PGE_2_ and PGF_2α_ are important in the multistage carcinogenic processes by sustaining epithelial hyperplasia, angiogenesis, immuno-suppression and tumor metastasis. But PGF_2α_ further has its distinct role in skin tumor promotion, angiogenesis, tumor invasion and metastasis [11]. PGF_2α_ may delay cell mitosis, inhibit cytokinesis, increase aneuploidy and genome instability, and thereby tumor promotion and cell transformation [11]. These results suggest a role of ANE-induced COX-2 expression and prostanoids production in mediating of oral cancer.

ANE can generate ROS during auto-oxidation in the oral cavity or by metabolism of intracellular enzymes [9], [25]. In this study, the ANE-induced COX-2 expression, PGE_2_ production and cytotoxicity to GK was prevented by catalase. This indicates ROS production may contribute to the ANE-induced inflammatory reaction of oral mucosa. ROS produced by ANE or generated by infiltrated inflammatory cells may also lead to genome instability of neoplastic cells, alter cellular signaling transduction and gene expression and promote malignant transformation [11], suggesting the use of antioxidants for prevention and treatment of BQ-chewing-related diseases. Little is known about HO-1 in oral carcinogenesis. Interestingly we found that ANE stimulated the expression of HO-1, an oxidative stress response gene with cytoprotective and inflammation resolution effect [23], suggesting the presence of adaptive response to ANE. The stimulation of HO-1 by ANE was mediated by ROS. HO-1 may catalyze the degradation of heme to form ferrous iron, carbon monoxide and biliverdin. Inhibition of HO-1 by Zn-protoporphyrin enhances the ANE cytotoxicity, revealing the protective effect of HO-1 against ANE.

Chemicals can generate ROS by auto-oxidation or via metabolic activation by enzymes such as cytochrome P450 (CYP) [4]. CYP are crucial molecules for tobacco-related oral carcinogenesis [26]. We proposed that CYP may involve in the metabolism of BQ components, transform procarcinogens to DNA-reactive intermediates and contribute to oral cancer. CYP is a group of enzymes (CYP1A1/1A2, CYP1B1, CYP2A6, CYP2D6, CYP2E1/F1 etc.) that can metabolize many distinct dietary or tobacco carcinogens with specificity. In this study, we found that GK express CYP1A1, 2C8/19, 2E1, 3A3/3A4, but little CYP1A2, 2B6/B7, 2A6/A7, 2F1, 3A5 and 3A7, indicating the involvement of CYPs in metabolizing specific endogenous and exogenous chemicals in oral epithelial cells. A decrease in CYP3A, an enzyme for oxidative metabolism of endogenous/exogenous substrates, elevated the risk of susceptibility to OSF [27]. A deficient in CYP2A6 may decrease the risk of oral cancer in BQ chewers in Sri Lanka [28]. We further found that ANE stimulated CYP1A1 expresssion in GK and α-naphthoflavone prevented the ANE-induced COX-2 expression and PGE_2_ production. This reveals that metabolism of ANE by CYPs is crucial for ANE-induced oral mucosal inflammation. However, α-naphthoflavone showed no significant protective effect on ANE-induced cytotoxicity. This indicates that metabolites generated from ANE by CYP1A1/1A2 activation may contribute to stimulation of COX-2 expression and PGE_2_ production, but not cytotoxicity. It is probable that additional ANE components are present and cause synergistically the cytotoxicity of ANE on GK. Accordingly, whilst CYP1A1 is the enzyme for metabolism of 2-acetylaminofluorene to generate electrophilic DNA-reactive metabolites, inhibition of CYP1A1 by α-naphthoflavone shows little effect on 2-acetylaminofluorene cytotoxicity toward liver epithelial cells [29]. Previous studies found that administration of ANE to mice stimulates hepatic CYP enzyme activities [30], but arecoline inhibits the 2,3,7,8-Tetrachlorodibenzo-p- dioxin (TCDD)-induced CYP1A1 expression in hepatoma cells [31]. Moreover the induction of CYP1A1 by ANE may potentially promote the metabolic activation of BQ-specific nitrosamines or other tobacco carcinogens and contribute to oral carcinogenesis [32].

BQ components may activate PI3K/Akt, MEK/ERK, JNK, p38 and NF-κB signaling to mediate COX-2 expression, PGE_2_ production and cell differentiation [12], [33], [34]. However, the upstream signaling responsible for initiation of these events is not clarified. EGFR, Src, and Ras are the upstream signaling molecules that are crucial for carcinogenesis [8], [35], [36]. EGF and EGFR modulate keratinocyte proliferation, migration and transformation that is important for wound healing and carcinogenesis. Previous reports have found an elevation of EGFR expression in OSCC and its association with the metastasis of OSCC [6], [13]. AG1478, an EGFR inhibitor, may inhibit tumor formation and progress in mouse OSCC models. ROS may activate EGFR signaling [37] and EGF can stimulate COX-2 expression via p38 and ERK, but independent of Src and NF-kB signaling in oral epithelial cells [38]. We further found that ANE activates EGFR (Tyr845) of GK to mediate COX-2 expression and PGE_2_ production. However, ANE does not activate EGFR (Tyr1068 and Tyr1173) in OECM-1 oral cancer cells [33]. The differential response of GK and OECM-1 cells to ANE is possibly due to difference in cell regulation of primary keratinocytes and cancer cell lines. ANE activates both ERK and JNK phosphorylation, whereas EGF stimulates ERK and p38 phosphorylation in OECM-1 cells [33]. Our results highlight the potential use of EGFR antibodies or antagonists for prevention or treatment of OSCC.

Src and Ras may regulate cell adhesion, invasion, proliferation, survival, and angiogenesis, which are crucial for tumor development [8], [35]. Various growth factors, receptor tyrosine kinase (e.g., EGFR) and Src may stimulate multiple signaling pathways such as Ras/MEK/ERK, GTPase activation proteins and focal adhesion kinases [8], [35]. We intriguingly found that ANE activates Src and Ras to mediate ANE-induced COX-2 expression and PGE_2_ production in primary GK. Sodium orthovanadate (a tyrosine phosphatase inhibitor) enhanced the ANE-induced PGE_2_ production in GK **(data not shown)**, further supporting the tyrosine kinase activation in mediating these events. Similarly, recent study has found that BQ ingredients may stimulate Src activation in oral cancer epithelial cells to promote cell migration and tumor invasion [39]. An elevated expression of Ras and mutation of Ras in oral premalignant lesions and OSCC of BQ chewers has been reported [14], [40]. This indicates that activation of Src and Ras is important in the BQ carcinogenesis.

Impairment of cell cycle control and aberrant epithelial differentiation are crucial for the initiation, promotion and progression of cancer. ANE may induce DNA breaks and stimulate differentiation of buccal epithelial cells as indicated by increased expression of involucrin [41]. However, ANE is recently shown to down-regulate involucrin expression in oral keratinocytes via PI3K/Akt but not MEK1/2 signaling [42]. The precise reasons are no clear. Keratin 5 and keratin 14 are two hallmarks expressed by mitotic active basal keratinocytes in the stratified squamous epithelium and have tissue protective function [43]. When the basal keratinocytes differentiate to suprabasal compartment, keratin 5 and keratin 14 expressions decreased [43]. The expression of keratins can be regulated at transcriptional level by AP-1, AP-2 or SP1. Thus expression keratin is important for the integrity and function of normal epithelium. In this study, we found that ANE inhibited the expression of keratin 5 and keratin 14, suggesting the induction of GK differentiation by ANE. The ANE-induced decline of keratin 14 expression is associated with CYP1A1 activation and Src signaling, but not directly via EGFR, Ras and COX activation. Src and PI3K signaling has been shown being activated in the early stage of epithelial differentiation [44]. Possibly the metabolism of ANE by CYP1A1 may stimulate multiple signaling pathways that show compensatory cross-talks. This may partly explain why an elevated expression of differentiation markers such as keratin 1 and involucrin was noted after exposure to tumor promoters and observed in most oral premalignant lesions, whereas a decreased expression is identified in severe dysplasia and poorly differentiated cancer [45].

We have found that ANE and arecoline may induce G2/M cell cycle arrest of oral epithelial cells via activation of Chk1/Chk2 signaling pathways to provide the time for DNA repair [17], [25], because ANE and arecoline are known to exhibit genotoxicity [2]. This may explain why ANE inhibits cdc25C and cyclin B1 expression of GK in this study. The inhibition of cdc25C and cyclin B1 was reversed by α–naphthoflavone and manumycin C, but not EGFR, Src and aspirin, indicating the involvement of CYP1A1 and Ras activation in this event. ANE may induce cell cycle arrest and cellular senescence with upregulation of p38, p21, p16, NF-κB, IL-6 and COX-2 in oral keratinocytes [34]. Ras may regulate p53 and further affect cdc25 and cyclinB1 and promote cellular senescence [46], revealing the Ras activation in modulation of oral carcinogenesis.

Clinically one critical health issue is whether addition of betel leaf (*piper betle* leaf, PBL) into BQ may enhance or attenuate the carcinogenicity and toxicity of BQ. We interestingly found that PBL extract and HC prevented the ANE-induced PGE_2_ production in GK. This reveals that suitable addition of PBL in BQ may provide some beneficial effects against ANE toxicity to tissue/organs. However, HC showed no marked effect on ANE-stimulated COX-2 expression, whilst PBL extract prevented the ANE-induced COX-2 expression. This suggests that effects by PBL are possibly related to inhibition of ANE-induced signaling transduction, gene expression at transcriptional/translational level and even enzyme activity. Accordingly HC can inhibit both COX-1 and COX-2 enzyme activities [16] and thereby COX-mediated products such as prostanoids and thromboxane in platelets.

In this study, GK was shown to express NQO-1 **(data not shown)**. Dicoumarol, a NQO-1 inhibitor, can attenuate the ANE-induced PGE_2_ production at concentrations of 10–20 µM. This is possibly due to its inhibition of NF-κB. But dicoumarol further enhanced the ANE-induced cytotoxicity at higher concentrations (50–100 µM), possibly due to its inhibition of NF-kB and NQO-1. Similarly, dicoumarol may enhance the 9–chloro-β-lapachone-induced cytotoxicity in hepatocytes [47]. Dicoumarol also enhanced the gemcitabine-induced cytotoxicity to cholangiocarcinoma cells [48]. NQO-1 has been shown to exhibit dual roles in detoxification or activation of procarcinogens. NQO-1 mediate the 2 electron reduction of quinone to hydroquinone for further conjugation and secretion but not the generation of toxic semi-quinone intermediates, thus reducing ROS production and electrophilic attack [48]. These results suggest that one electron reduction of quinone by NADPH-CYP450 reductase may generate more toxic semiquinone intermediates, whereas 2 electron reduction of ANE components by NQO-1 generate less cytotoxic intermediates. The other possibility is inhibition of NQO-1 promote the cytotoxicity of ANE components with quinone-like structure that is widely distributed in natural products and clinical drugs. Accordingly ANE has been shown to contain polyphenols and catechols with pro-oxidant property and quinine structure that can be metabolized by CYP and NQO-1 to affect their cytotoxicity especially in the presence of transition metals [49]. AN polyphenols have been shown to inhibit COX-2 expression at low concentration, but stimulate COX-2 at higher concentrations [50]. Since catechol cannot stimulate PGE_2_ production of GK in our study, this indicates that semiquinone intermediates generated during metabolism of AN polyphenols and catechol may contribute to ANE-induced cytotoxicity to GK.

**Figure 7:**
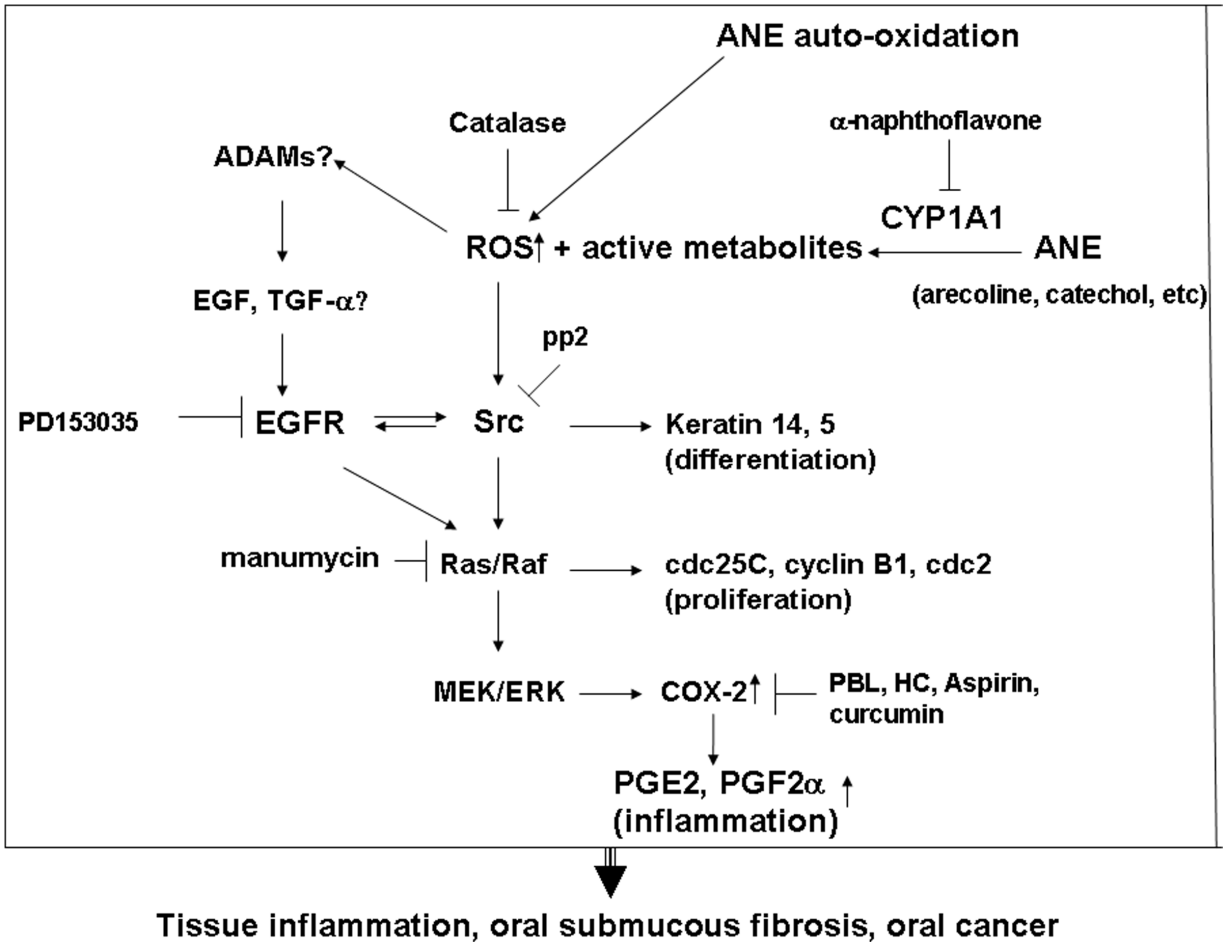
The proposed mechanism of ANE-induced molecular changes (CYP1A1, ROS, Src, EGFR, Ras, COX, PGE_2_, keratins and cyclin B1 etc.) in oral mucosal cells. Signal transduction pathways responsible for the AN-induced alterations in cell proliferation, differentiation and inflammation of GK were shown.

In conclusion, auto-oxidation or metabolic activation of ANE components by CYP1A1 may generate ROS and reactive metabolites **(** **Figure 7** **)**. ROS could activate multiple signaling pathways such as EGFR, Src and Ras to affect cdc25C, cyclin B1 and keratin 14 expression, to mediate MEK/ERK signaling, COX-2 expression and prostanoids production. PBL extract, HC and curcumin inhibited the ANE-induced PGE_2_ production by differential mechanisms. The aberrant growth, differentiation and inflammation induced by BQ components may contribute to the pathogenesis of OSF and OSCC. Future targeting therapy for EGFR, Src, and Ras may be potentially used clinically for prevention and treatment of BQ-chewing-related diseases. Moreover, the addition of PBL (with HC) into BQ can potentially attenuate the oral mucosal inflammatory response. Dietary consumption or intake of curcumin is a useful strategy to prevent the BQ-chewing-related mucosal inflammatory response and even carcinogenesis. Whether BQ components may activate upstream a disintegrin and metalloproteinases (ADAMs) and EGF/TGF production should be further addressed to clarify the pathogenesis of BQ carcinogenesis.
